# La maladie de Wilson chez l'enfant: à propos de 20 cas

**DOI:** 10.11604/pamj.2013.14.6.995

**Published:** 2013-01-03

**Authors:** Mounia Lakhdar Idrissi, Abdeladim Babakhoya, Kawtar Khabbache, Fatimzohra Souilmi, Sara Benmiloud, Sanae Abourrazak, Sanae Chaouki, Samir Atmani, Abdelhak Bouharrou, Moustapha Hida

**Affiliations:** 1 Service de pédiatrie, CHU Hassan II, Fès, Maroc

**Keywords:** Enfant, hépatopathie, système nerveux central, Wilson, Child, hepatopathy, central nervous system, Wilson disease

## Abstract

La maladie de Wilson ou dégénérescence hépato-lenticulaire est une affection génétique autosomique récessive caractérisée par une accumulation toxique de cuivre dans l'organisme, essentiellement dans le foie, le système nerveux central et la cornée. L'objectif de ce travail était de soulever les difficultés diagnostiques et thérapeutiques dans la prise en charge de la maladie de Wilson dans notre contexte. Nous avons réalisé une étude rétrospective portant sur 20 cas de maladie de Wilson colligés au sein du service de pédiatrie du CHU HASSAN II de Fès sur une période de 7 ans et demi. Il s'agit de 13 garçons et 7 filles dont l’âge moyen est de 9 ans avec des extrêmes allant de 5 à 13 ans. La consanguinité est retrouvée chez 13 malades. Sur le plan clinique, l'ictère est noté dans 13 cas, un syndrome oedémateux est retrouvé dans 13 cas aussi et un syndrome hémorragique dans 6 cas. Les signes neurologiques sont présents dans 7cas. Trois enfants étaient asymptomatiques diagnostiqués à l'occasion d'un dépistage. Sur le plan biologique les signes d'insuffisance hépatocellulaire sont retrouvés chez 17 malades avec une cytolyse dans 8 cas. Une anémie hémolytique est retrouvée chez 8 malades (soit 40%). La céruléoplasminémie est abaissée chez 17 malades, la cuprurie réalisée chez 19 malades s'est révélée augmentée chez 17 soit 89,4%. L'anneau de Kayser- Fleischer est retrouvé chez 14 patients. L’échographie abdominale a montré des signes d'hypertension portale (HTP) sur foie de cirrhose chez 16 malades soit 80%. La D pénicillamine est instaurée chez 17 patients et trois sont mis sous sulfate de zinc. Trois malades ont bénéficié de la vitamine B6. L’évolution est favorable chez 11 malades avec un recul moyen de 3 ans. Nous déplorons 4 décès chez des malades ayant consulté au stade de cirrhose décompensé. Le pronostic de la maladie de Wilson dépend de la précocité du traitement. Le dépistage chez les membres de la famille est une démarche importante et obligatoire pour un diagnostic précoce.

## Introduction

La maladie de Wilson est une maladie rare. On estime qu'il y a un enfant malade pour 30000 naissances. La maladie se développe le plus souvent dans la première ou deuxième décennie de la vie. La présentation clinique de cette maladie est caractérisée par une très grande hétérogénéité de symptômes. Ces manifestations peuvent rester isolées, s'associer ou se succéder expliquant la variété des tableaux cliniques rencontrés. Si son diagnostic est fait précocement, il s'agit de l'une des maladies héréditaires les plus faciles à traiter. En l'absence de tout traitement, l’évolution spontanée est le plus souvent mortelle. Actuellement plusieurs options thérapeutiques sont disponibles pour stabiliser la maladie et éviter les dommages liés à l'accumulation du cuivre sous réserve d'une bonne observance thérapeutique. Le but de notre travail est d’étudier les caractéristiques de la maladie de Wilson tant sur le plan clinique, para clinique, thérapeutique et évolutif tout en discutant les différentes difficultés diagnostiques et thérapeutiques rencontrées dans notre pratique.

## Méthodes

Nous avons réalisé une étude prospective portant sur 20 enfants suivis dans le service de pédiatrie du Centre Hospitalier et Universitaire Hassan II de Fès sur une période de 7ans et demi (2003 - 2010). Ont été inclus les patient ayant un âge moins de 16 ans, une atteinte hépatique avec ou sans atteinte neurologique, un bilan cuprique perturbé et/ou la présence d'un anneau de Keyser-Fleischer. Nous avons analysé les paramètres cliniques, biologiques (hépatiques et cupriques) et évolutifs. L'enquête familiale à la recherche de cas similaires a été faite par l'examen clinique et l'analyse du bilan cuprique de la fratrie.

## Résultats

L’âge moyen de découverte de la maladie de Wilson dans notre série était de 9 ans avec des extrêmes de 5 et 13 ans. Le sexe masculin était plus touché avec 13 cas sur 20 soit 65%. Le sexe ratio (M /F) était donc de 1,8.

La consanguinité a été retrouvée chez 13 patients (soit 65%). Dix d'entre eux avaient une consanguinité de premier degré, les trois autres avaient une consanguinité de deuxième degré. Dans les antécédents de nos malades, on a noté la notion de décès dans la fratrie dans un tableau similaire dans sept cas. Le délai moyen entre l'apparition des premiers signes cliniques et la consultation a été de 5 mois. Les signes cliniques à l'admission étaient dominés par le tableau d'HTP avec un syndrome oedémateux et un ictère dans 65% des cas; une hépatomégalie était retrouvée dans 6 cas et une splénomégalie dans 12 cas. L'examen neurologique a révélé l'existence d'un syndrome extrapyramidal dans 4 cas. Un seul patient avait présenté des arthralgies et 3 patients était asymptomatiques diagnostiqués lors du dépistage dans la fratrie des malades de notre série. Les signes cliniques sont rapportés sur le Tableau 1. L'examen ophtalmologique à la lampe à fente a mis en évidence la présence de l'anneau de Kayser-Fleischer chez 14 malades soit 70%.

**Tableau 1:** Les signes cliniques retrouvés dans notre série

Les signes cliniques	Nombre	Pourcentage
Ictère	13	65
Syndrome oedémateux	13	65
Hépatomégalie	6	30
Splénomégalie	12	60
Syndrome hémorragique	6	30
**Signes neurologiques**		
Dysarthrie et hypersialorrhée	3	15
Dysarthrie et hypersialorrhée	3	15
Dystonie	1	5
Arthralgies	1	5
Asymptomatique	3	15

Biologiquement, les transaminases étaient normales chez 12 malades soit 60%, modérément élevées chez 6 soit 30% et très élevées chez 2 soit 10%. Le TP était inférieur à 40% chez 9 malades (soit 45% des cas), entre 40% et 70% chez 8, alors qu'il était normal chez les 3 autres. La bilirubinémie totale élevée dans 13 cas avec prédominance de bilirubine libre dans 50% des cas. L'EPP réalisée chez 15 malades, a montré une hypoalbuminémie chez 10 et une hypergammaglobulinémie dans 14 cas. Sur le plan hématologique une anémie hémolytique a été retrouvée chez 8 malades (40%) et une thrombopénie chez 13 malades (65%).

**Figure 1:**
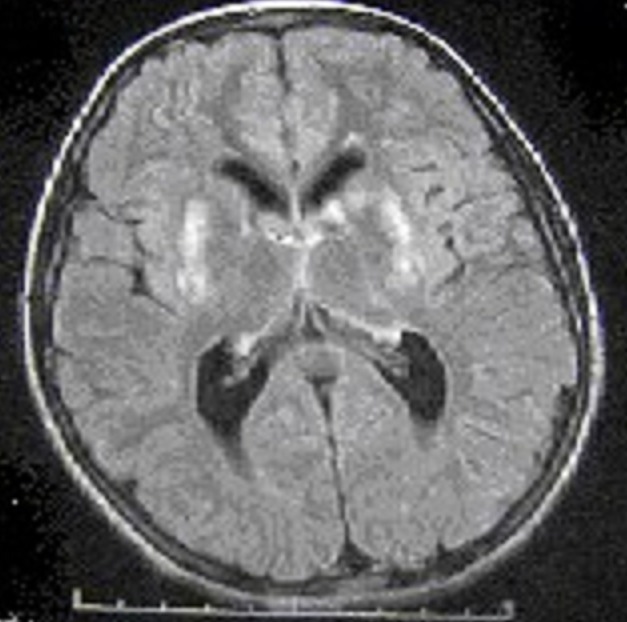
IRM d'un patient montrant un hyper signal flair des 2 putamens

La céruloplasminémie est abaissée chez 17 malades, soit 85%, et normale chez les 3 autres. La cuprémie est abaissée chez 8 patients, soit 40%, normale chez deux et augmentée chez un seul malade. La cuprurie réalisée chez 19 malades s'est révélée augmentée chez 17 soit 89% (Tableau 2). L’échographie abdominale a montré des signes d'HTP sur foie de cirrhose chez 16 malades soit 80%. La fibroscopie digestive a objectivé des varices oesophagiennes chez 12 malades; elles sont de stade 1 dans 6 cas, de stade 1 à 2 dans 3 cas et de stade 3 dans 3 cas. L'IRM n'a été réalisée que chez 3 malades qui avaient des signes neurologiques. Elle avait mis en évidence pour les 3 cas un hyper signal T2 et flair des 2 putamens (Figure 1:). Aucune étude moléculaire n'a pu être réalisée chez nos malades.

**Tableau 1:** Le bilan cuprique de nos malades

Bilan cuprique (sur le nombre de malades)	Basse	Normale	Élevée
Céruloplasminémie (sur 20)	17 (85%)	3	0
cuprurie (sur 19)	0	2	17 (89%)
Cuprémie (sur 11)	8 (40%)	2	1

Sur le plan thérapeutique 17 de nos malades ont été traités par la D pénicillamine (TROLOVOL^®^) et trois par le sulfate de zinc (ZINASKIN^®^). Trois malades ont bénéficié de la vitamine B6.

Après un recul moyen de 3 ans avec des extrêmes de 4 mois à 5 ans et demi, l’évolution était marquée sous traitement par une stabilisation clinique et biologique chez 12 malades. On a déploré 4 décès dans un tableau de cirrhose décompensée chez des patients ayant consulté tardivement pour un syndrome oedemato-ascitique, ictère et insuffisance hépato-cellulaire. Chez 4 patients, l’évolution n'a pu être appréciée car ils étaient perdus de vue.

## Discussion

La maladie de Wilson (MW) ou “dégénérescence hépato-lenticulaire’ est une affection génétique de transmission autosomique récessive. Il s'agit d'une toxicose cuprique caractérisée par une accumulation tissulaire de cuivre libre: essentiellement hépatique, cérébrale et péricornéenne. Cette maladie résulte des mutations du gène de l'ATP7B porté par le chromosome 13. Cette protéine ATP7B assure le transport du cuivre dans l'hépatocyte. En France la prévalence des homozygotes dans la population générale est de l'ordre de 30/1.000.000 [1]. La maladie de Wilson peut être observée dans toutes les ethnies; la consanguinité augmente considérablement son incidence. De ce fait, elle serait assez fréquentes dans les pays de Maghreb vue la fréquence des mariages consanguins. Son incidence exacte dans ces pays n'est pas connue. Au Maroc, elle reste sous-estimée du fait de la difficulté que pose son diagnostic.

Sur le plan clinique, la MW se manifeste exceptionnellement avant l’âge de 3 ans reflétant probablement les capacités considérables du foie à stocker l'excès du cuivre. Dans la majorité des cas, les symptômes apparaissent entre 5 et 10 ans, et occasionnellement peuvent survenir après 50 ans [2]. Les manifestations cliniques peuvent se présenter sous diverses formes et sont très hétérogènes tant dans leur présentation que dans la date d'apparition des premiers troubles. La maladie se révèle chez 45% des patients par une symptomatologie liée à l'atteinte hépatique, chez 35% des patients par des signes neurologiques et dans 10% par des troubles psychiatriques. Dans les autres cas les manifestations sont hématologiques, rénales ou ostéoarticulaires [3].

Les manifestations hépatiques prédominent chez l'enfant alors que les manifestations neurologiques sont plus fréquentes au-delà de 18 ans. Certains auteurs rapportent que les sujets dont la maladie s'est révélée par des manifestations hépatiques sont généralement plus jeunes que ceux dont les premiers symptômes sont neuropsychiatriques et que les formes à présentation hépatique ont un risque de décès cinq fois plus élevé que les formes à présentation neurologique [4]. Dans notre étude, les premières manifestations sont hépatiques chez 17 malades soit 85% et neurologiques chez 3 malades soit 15%. L'atteinte hépatique est souvent isolée chez l'enfant, découverte entre 5 et 13 ans. Elle peut aller d'une simple augmentation des transaminases à une hépatite fulminante. Il convient d’évoquer l'hypothèse d'une maladie de Wilson devant toute hépatopathie chronique non virale et non toxique chez l'enfant de plus de 5 ans. Plusieurs tableaux cliniques peuvent être révélateurs: hépato-splénomégalie associée ou non à des angiomes stellaires et à des anomalies du bilan biologique; hépatopathie aigue régressive sans caractéristique clinique particulière mais dans laquelle l'attention sera alerté par la présence d'une anémie hémolytique, d'une élévation de la bilirubine libre, d'un abaissement de l'acide urique; hépatopathie fulminante dont le taux de mortalité est très élevé; hépatopathie chronique active, dans 10 à 30% des cas; cirrhose progressive avec splénomégalie plus que l'hépatomégalie, signes d'hypertension portale et encéphalopathie éventuelle [5, 6]. Dans notre série la majorité de nos malades se sont présentés d'emblée dans un tableau de cirrhose ce qui suggère un retard de diagnostic dû le plus souvent à un retard de consultation mais aussi à cette hétérogénéité clinique qui rend difficile l'hypothèse diagnostique. Certains patients peuvent même décéder avant que le diagnostic ne soit posé. Le retard diagnostique peut aussi être dû à une évolution rapidement sévère puisque le délai moyen d’évolution retrouvé était de 5 mois. Les mutations présentes dans notre population seraient-elles différentes de celles déjà décrites. ‘Un travail déterminant le profil génétique de nos malades est indispensable.

L'atteinte neurologique dans la MW est au début souvent discrète, mais doit être systématiquement recherchée car elle constitue un appoint pour le diagnostic. Son installation brutale après un facteur déclenchant tel un traumatisme ou surtout une intervention chirurgicale sous anesthésie générale est décrite. Elle s'observe entre 15 et 30 ans, elle est exceptionnelle avant l’âge de 12 ans. Elle peut se manifester par des signes frustres [7, 8]. Il s'agit en effet d'un retard des acquisitions scolaires, de troubles de comportement à type notamment de trouble de l'humeur. Peu à peu apparaissent des tremblements, une démarche lente, une mimique pauvre avec faciès inexpressif et écoulement salivaire, une voix monocorde, des mouvements athétosiques et/ou choréiques vont compléter le tableau et s'aggraver par les stimulations et les émotions. Les mouvements choréiques peuvent orienter à tort vers une chorée de Sydenham. Le tableau neurologique se complète ensuite, dominé par la rigidité et les mouvements anormaux. La MW peut aussi se révéler par un torticolis; l'aspect atypique et l’évolution oriente le diagnostic [9]. Les troubles psychiatriques sont inauguraux dans 15% des cas ou accompagnent les manifestations neurologiques. Ils sont alors d'autant plus sévères que la symptomatologie neurologique est avancée [10].

Les manifestations ophtalmologiques usuelles de la MW sont la présence d'anneau vert péricornéen de Kayser-Fleischer qui reflète le dépôt de cuivre dans la membrane de Descemet. Il est soigneusement recherché à la lampe à fente par un ophtalmologiste expérimenté et est quasi pathognomonique de la maladie. Mais il peut être observé en cas de choléstase importante, notamment en cas de cirrhose biliaire primitive ou cryptogénique et en cas de cholangite sclérosante primitive [11]. Chez les patients atteints de troubles neurologiques, il est presque toujours présent. Mais dans les formes hépatiques, il peut être absent dans 25% des cas. S'il n'est pas retrouvé, on ne peut donc exclure le diagnostic de la MW. Dans notre série, il était présent dans 70% des cas.

Une anémie hémolytique à test de Coombs négatif, aigue ou subaigüe, est parfois rencontrée comme présentation inaugurale. La plupart des cas ont une cirrhose avec une anémie hémolytique à bas bruit, chronique, avec quelques épisodes aigus qui peuvent précéder la symptomatologie hépatique ou neurologique de quelques années [12].

La thrombopénie est secondaire d'une part à l'hypersplénisme et d'autre part à la toxicité directe du cuivre. La cirrhose engendre un hypersplénisme qui séquestre tous les éléments figurés du sang dont les plaquettes. Il y a donc une accélération de la destruction plaquettaire. Par ailleurs, le cuivre intervient sur la mégacaryogénèse en provoquant une anomalie de la production plaquettaire.

L'atteinte rénale est quasiment constante mais reste le plus souvent latente. Les manifestations rénales sont le plus souvent liées à des lésions tubulaires proximales, beaucoup plus rarement à une atteinte glomérulaire. Dans tous les cas, elles sont secondaires à la toxicité du cuivre [13].

Les formes asymptomatiques sont habituellement dépistées, idéalement vers l’âge de 3 à 4 ans, dans la fratrie d'un enfant chez qui une MW vient d’être diagnostiquée. Les signes biologiques se résument à une élévation modérée et persistante des transaminases, mais celle-ci peut être absente alors que la balance cuprique est positive depuis de nombreuses années. L'apport diagnostique de la biologie moléculaire est ici important lorsque l’étude chez la fratrie s'est révélée informative.

Les perturbations biologiques spécifiques de la MW reposent sur l'analyse du bilan cuprique. La céruloplasminémie est effondrée chez environ 80% des patients ce qui est compatible avec notre série. Dans 10 à 15% des cas, son taux sera intermédiaire. Cependant, chez 5 à 10% des patients, ce taux peut approcher la normale ou même être normal. Par ailleurs, jusqu’à 20% des porteurs homozygotes ont un taux bas de céruloplasmine malgré le fait qu'ils ne développeront pas la maladie. Le taux de céruloplasmine peut donc constituer un facteur de suspicion mais pas de diagnostic de la MW. Dans notre série la céruloplasmine était abaissée chez 85% des malades.

Le cuivre sérique est en général très diminué en cas de maladie de Wilson. La cuprémie totale est constituée par le cuivre lié à la céruloplasmine (92%) et le cuivre libre ionique. La cuprémie totale est en principe basse mais non effondrée car il y a une augmentation de la fraction libre du cuivre. Ce dosage est souvent très variable au cours de la maladie et donc peu d'utilité en pratique.

Le dosage du cuivre urinaire est indispensable au diagnostic de la MW. Chez les patients symptomatiques, le taux est systématiquement élevé (>100 ‘g/24 h) si la totalité des urines de 24 heures a été prélevée et si les urines sont exemptes d'une contamination par le cuivre. Ce taux doit être confirmé sur des prélèvements des urines de 24 heures répétés une ou deux fois, car il existe une variabilité d'un jour à l'autre influencé en particulier par l'alimentation. Une seule autre situation peut présenter une élévation du cuivre urinaire: une affection hépatique obstructive telle une cirrhose biliaire primitive ou une choléstase. Dans cette situation, les taux du cuivre hépatique et urinaire peuvent être élevés, et même l'anneau de Kayser-Fleischer peut être présent. Un taux de cuivre urinaire < 50 ‘g/24h chez un patient symptomatique et en absence d'insuffisance rénale, exclut pratiquement le diagnostic de la MW. Si la valeur du cuivre urinaire est comprise entre 50 et 100 ‘g/24 h, des investigations complémentaires sont justifiées. Des valeurs à la limite supérieure de la normale chez des sujets présymptomatiques ne doivent pas exclure le diagnostic dans la fratrie des patients affectés. Ce dosage est aussi extrêmement important pour suivre l'efficacité et l'observance du traitement [14].

La deuxième difficulté diagnostique qui se révèle dans notre contexte est due au problème de réalisation du bilan cuprique qui n'est pas fait de façon routinière dans nos laboratoires et de ce fait il est souvent très cher pour une population de revenue limité; ceci d'autant plus que le même bilan est demandé pour le dépistage de tous les membres de la fratrie. Enfin, l'interprétation de ce bilan n'aboutit pas toujours à un diagnostic de certitude [15]. En effet, de rares homozygotes malades peuvent avoir des taux en cuivre et en céruloplasmine quasiment normaux [16]. La céruloplasmine peut même être élevée dans certains cas. Des hétérozygotes en principe indemnes peuvent avoir une cuprémie et un taux de céruloplasmine très abaissés. D'autre part, seul le dosage de la cuprèmie totale est disponible, et l'hypercuprurie, constante dans les formes neurologiques, ne l'est pas dans les formes hépatiques qui sont plus fréquentes chez nous. Or, il est important d’établir un diagnostic de certitude; d'une part pour les hétérozygotes qui ne doivent pas être traités inutilement, et d'autre part pour les homozygotes atteints, chez qui un traitement précoce pourra éviter la survenue de signes cliniques. Les autres tests, de grand apport diagnostique, ne sont pas de pratique courante; il s'agit du dosage du cuivre hépatique par biopsie (> 250 ‘g/g de tissu sec) qui permet aussi une surveillance de l'efficacité du traitement, les tests d'incorporation du cuivre marqué et la biologie moléculaire qui est la plus spécifique. Aujourd'hui, deux stratégies d’étude génétique sont utilisées, soit le diagnostic familial indirect par analyse d'haplotypes ou le diagnostic direct par recherche de mutation [17, 18]. Malheureusement ces techniques ne sont pas disponibles dans notre pays.

Dans les formes neurologiques, la tomodensitomérie cérébrale peut être normale, même quand l'anneau de Kayser-Fleischer est présent. Dans la majorité des cas, elle montre une hypodensité bilatérale caractéristique des noyaux lenticulaires. En revanche, l'IRM est presque toujours pathologique dans les formes neurologiques et révèle des hyper signaux des noyaux gris de taille et de forme variables sur les séquences pondérées en T2. L'imagerie cérébrale est aussi utile pour suivre l’évolution des lésions sous traitement.

### Traitement

La MW est parmi les rares maladies métaboliques traitables. Son traitement doit être instauré à vie et précocement car permet une réversibilité des déficits non définitifs. Une fois les dommages irréversibles installés, l'effet du traitement est limité et la qualité de vie du patient est définitivement compromise. Ce traitement comporte un régime alimentaire évitant les aliments riches en cuivre (éviter champignons, foie, fruits de mer, noix, chocolat). Les chélateurs du cuivre sont l'essentiel du traitement. Ce sont la D-pénicillamine et la Trientine. Les inhibiteurs de l'absorption digestive du cuivre (sulfates de Zinc essentiellement et le tétramolybdate d'ammonium secondairement) représentent une autre approche thérapeutique possible [13]. Le suivi du traitement doit être clinique et biologique (cuprurie). L'efficacité de la prise en charge thérapeutique n'est observée que plusieurs mois après son introduction, notamment par la normalisation du bilan hépatique. Dans les formes neurologiques, l'amorce du traitement peut d'une aggravation du tableau moteur, aggravation suivie d'une amélioration secondaire pouvant ne pas permettre de regagner l’état neurologique pré thérapeutique. La greffe hépatique peut être envisagée en cas d'hépatite fulminante [11]. Son intérêt dans les formes neurologiques graves, résistantes au traitement médical bien conduit, est insuffisamment évalué. La discussion reste ouverte sur les avantages et les inconvénients du zinc et de la D-Pénicillamine en première intention. Pour les deux traitements, ont été rapportés des échecs ou des aggravations neurologiques initiales mais des études récentes montrent une plus grande efficacité des traitements chélateurs par rapport aux sulfates de zinc dans la prévention de la détérioration hépatique [19]. Dans les formes présymptomatiques ou paucisymptomatiques, on s'accorde à traiter par le zinc, peu coûteux et ayant peu d'effets secondaires [20].

La difficulté dans notre pays est celle de se procurer la D-pénicillamine puisque ce produit n'est pas disponible au Maroc. Les décès recensés dans notre série sont dus soit à un retard ou un défaut de diagnostic soit à un retard de mise sous traitement ou à une mauvaise observance thérapeutique.

## Conclusion

Le pronostic de la maladie de Wilson apparaît d'autant meilleur que les atteintes neurologique et hépatique sont peu prononcées. La précocité du diagnostic représente donc un élément capital pour un bon pronostic de la maladie. L'idéal étant d'affirmer le diagnostic à son stade présymptomatique par le dépistage familial.
